# Device and surgical procedure-related infections in Canadian acute care hospitals, 2019–2023

**DOI:** 10.14745/ccdr.v51i67a05

**Published:** 2025-07-01

**Authors:** Canadian Nosocomial Infection Surveillance Program

**Affiliations:** 1 Centre for Communicable Diseases and Infection Control, Public Health Agency of Canada, Ottawa, ON

**Keywords:** hospital-associated infection, acute care, surveillance, antimicrobial resistance, device-associated infection, surgical procedure-related infection, surgical site infection, ICU-CLABSI, central line-associated bloodstream infection, hip and knee arthroplasty surgical site infection, cerebrospinal fluid shunt surgical site infection, paediatric cardiac surgical site infection, Canada

## Abstract

**Background:**

Healthcare-associated infections (HAIs) are a significant healthcare burden in Canada. National surveillance of HAIs at sentinel acute care hospitals is conducted by the Canadian Nosocomial Infection Surveillance Program.

**Objective:**

This article describes device and surgical procedure-related HAI epidemiology in Canada from 2019 to 2023.

**Methods:**

Data were collected from 68 Canadian sentinel acute care hospitals between January 1, 2019, and December 31, 2023, for intensive care unit central line-associated bloodstream infections (ICU-CLABSIs), hip and knee surgical site infections (SSIs), cerebrospinal fluid (CSF) shunt SSIs and paediatric cardiac SSIs. Case counts, rates, patient and hospital characteristics, pathogen distributions and antimicrobial resistance data are presented.

**Results:**

Between 2019 and 2023, 2,582 device-related infections and 1,029 surgical procedure-related infections were reported. Rates of ICU-CLABSIs fluctuated throughout the study period, with an overall increase in all intensive care unit settings except for the neonatal intensive care unit, where a 4% decrease was noted. An increase in SSIs following knee arthroplasty was observed, rising from 0.34 to 0.43 infections per 100 surgeries. Fluctuating trends were also observed in CSF shunt SSIs and paediatric cardiac SSIs over the study period. The most commonly identified pathogens were coagulase-negative staphylococci (23%) in ICU-CLABSIs and *Staphylococcus aureus* (42%) in SSIs.

**Conclusion:**

Epidemiological and microbiological trends among selected device and surgical procedure-related HAIs are essential for benchmarking infection rates nationally and internationally, identifying any changes in infection rates or antimicrobial resistance patterns and helping inform hospital infection prevention and control and antimicrobial stewardship policies and programs.

## Introduction

Healthcare-associated infections (HAIs) are a common outcome of healthcare delivery, impacting patient morbidity and mortality, increasing the burden on hospitals and costs, and contributing to the rise of antimicrobial resistance ((1)). Healthcare-associated infections can result from various factors, including the use of invasive medical devices and surgical procedures ((2)). Surgical site infections (SSIs) are one of the most common HAIs reported in hospitals and are associated with an increased hospital length of stay, as well as higher intensive care unit (ICU) admissions and hospital readmissions ((3)). Device and surgical procedure-related infections are also associated with a high-financial burden on the healthcare system accounting for almost $50,000 per central line-associated bloodstream infection (CLABSI) case and $28,000 per SSI case ((4)).

A point prevalence study conducted in 2017 in Canadian sentinel acute care hospitals revealed that 35.6% of all reported HAIs were linked to medical devices and surgical procedures ((5)). Among these, ICU-CLABSIs represented 21.2%, while SSIs associated with a prosthetic implant accounted for 19.4% ((5)). The risk of device and surgical procedure-related infections is associated with patient demographics and comorbidities, in addition to the type of hospital in which the patient received care ((6–8)).

Understanding the epidemiology of HAIs related to medical devices and surgical procedures is crucial for establishing benchmark rates over time. These benchmarks support the development of effective antimicrobial stewardship programs and guide infection prevention and control strategies. Collecting and analyzing antimicrobial susceptibility data are crucial for guiding appropriate antimicrobial use and combating antimicrobial resistance ((9)). This report presents an epidemiological summary of specific device- and surgical procedure-related HAIs reported between 2019 and 2023 across 68 hospitals participating in the Canadian Nosocomial Infection Surveillance Program (CNISP).

## Methods

### Design

Since its establishment in 1994, CNISP has conducted national HAI surveillance at sentinel acute care hospitals across Canada, in collaboration with the Public Health Agency of Canada and the Association of Medical Microbiology and Infectious Disease Canada. Data are presented for the following device and surgical procedure-related HAIs: ICU-CLABSIs; hip and knee arthroplasty SSIs; cerebrospinal fluid (CSF) shunt SSIs; and paediatric cardiac SSIs.

### Case definitions

Device and surgical procedure-related HAIs were defined according to standardized protocols and case definitions (see **Appendix**). Complex infections, defined as deep incisional and organ/space, were included in hip and knee SSI surveillance, while CLABSIs identified in ICU settings were included in CLABSI surveillance. The adult mixed patient ICU, adult cardiovascular surgery intensive care unit (CVICU), paediatric intensive care unit (PICU) and neonatal intensive care unit (NICU) were included as eligible ICU settings. Adult mixed ICUs included any adult ICU with a mix of patient types as part of the ICU patient mix (i.e., medical/surgical, surgical/trauma, burn/trauma, medical/neurosurgical).

### Data source

Epidemiological data for device and surgical procedure-related infections identified between January 1, 2019, and December 31, 2023 (using surgery date for SSIs and date of positive blood culture for CLABSIs) were submitted by participating hospitals using standardized data collection forms. Hospital participation varied by surveillance project and year. Data submission and case identification were supported by training sessions and periodic evaluations of data quality.

### Statistical analysis

To calculate hip and knee SSI, CSF shunt SSI and paediatric cardiac SSI rates, the number of cases were divided by the number of surgical procedures performed (multiplied by 100). To calculate ICU-CLABSI rates, the number of cases was divided by line day denominators (multiplied by 1,000). Neonatal ICU CLABSI rates stratified by birth weight category were not included in this report. To calculate ICU-specific catheter utilization, the total number of ICU patient central line days was divided by the total number of ICU patient days. To calculate proportions of pathogens, the number of pathogens were divided by the total number of identified pathogens. Denominators may vary, as missing and incomplete data were excluded from analyses. Median and interquartile ranges (IQR) were calculated for continuous variables. Trends over time were tested using the Mann-Kendall test. The chi-square test was used to compare two categorical variables. Significance testing was two-tailed and differences were considered significant at a *p*-value of ≤0.05. Analyses were conducted using R version 4.3.2.

## Results

Sixty-eight hospitals submitted device and surgical procedure-related infection data to CNISP between 2019 and 2023 (**Table 1**), with medium-sized (n=201−499 beds) mixed hospitals (n=15 sites, 22%) being the most common (data not shown). Overall, 2,582 device-related infections and 1,029 surgical procedure-related infections were reported. Among all SSIs reported (n=1,029), hip and knee infections represented 67% (n=694) of these types of infections.

**Table 1:** Characteristics of acute care hospitals participating in device and surgical procedure-related healthcare-associated infection surveillance, 2023

Characteristic of hospitals	CLABSI-adult mixed ICU	CLABSI-adult CVICU	CLABSI-PICU	CLABSI-NICU	CSF shunt SSI	Paediatric cardiac SSI	Hip and knee SSI	Total unique hospitals
Total number of participating hospitals	40	9	12	20	13	6	31	68
**Hospital type**								
Adult^a^	27	6	N/A	3	4	N/A	15	34
Mixed^b^	13	3	4	9	1	N/A	16	25
Paediatric^c^	N/A	N/A	8	8	8	6	N/A	9
**Hospital size**								
Small (1–200 beds)	4	1	7	4	7	4	6	20
Medium (201–499 beds)	21	3	4	7	3	2	14	30
Large (500 and more beds)	15	5	1	9	3	0	11	18

Abbreviations: CLABSI, central line-associated bloodstream infection; CSF shunt SSI, cerebrospinal fluid shunt surgical site infection; CVICU, cardiovascular surgery intensive care unit; ICU, intensive care unit; N/A, not applicable; NICU, neonatal intensive care unit; PICU, paediatric intensive care unit; SSI, surgical site infection

^a^ Three hospitals classified as “adult” also had a neonatal intensive care unit

^b^ Mixed hospitals provide both adult and paediatric care

^c^ Paediatric standalone hospitals excluding mixed facilities with women’s and obstetric wards

A total of 2,854 pathogens were identified from device-related infections and 1,061 pathogens from surgical procedure-related cases between 2019 and 2023. Of the identified pathogens for ICU-CLABSIs, 60% were gram-positive, 25% were gram-negative and 16% were fungal. Of the identified pathogens for SSIs, 80% were gram-positive, 19% were gram-negative and 1% were fungal. Coagulase-negative staphylococci (CoNS) and *Staphylococcus aureus* were the most common pathogens associated with SSIs, while CoNS and *Enterococcus* spp. were most frequently identified in cases of ICU-CLABSIs, respectively (**Table 2**). From 2019 to 2023, the proportion of methicillin-resistant *S. aureus* (MRSA) was 14% for ICU-CLABSIs and 11% for SSIs (data not shown).

**Table 2:** Distribution and rank of the most frequently reported gram-negative, gram-positive and fungal pathogens, 2019–2023^a^

Pathogen category	Rank	Pathogen	ICU-CLABSI		Hip and knee		CSF shunt		Paediatric cardiac	
			N=2,854		N=780		N=130		N=151	
			n	%	n	%	n	%	n	%
Gram-positive	1	Coagulase-negative staphylococci^b^	661	23.2	134	17.2	46	35.4	20	13.2
	2	*Enterococcus* spp.	590	20.7	34	4.4	3	2.3	0	0.0
	3	*Staphylococcus aureus* ^c^	273	9.6	317	40.6	37	28.5	93	61.6
	4	*Streptococcus* spp.	60	2.1	81	10.4	4	3.1	10	6.6
	Other gram-positive^d^		124	4.3	59	7.6	12	9.2	2	1.3
	Total gram-positive		1,708	59.8	625	80.1	102	78.5	125	82.8
Gram-negative	1	*Klebsiella* spp.	159	5.6	14	1.8	8	6.2	5	3.3
	2	*Escherichia coli*	130	4.6	26	3.3	7	5.4	1	0.7
	3	*Enterobacter* spp.	110	3.9	33	4.2	4	3.1	6	4.0
	4	*Pseudomonas* spp.	82	2.9	28	3.6	3	2.3	2	1.3
	5	*Serratia* spp.	58	2.0	9	1.2	1	0.8	1	0.7
	Other gram-negative^e^		163	5.7	43	5.5	4	3.1	3	2.0
	Total gram-negative		702	24.6	153	19.6	27	20.8	18	11.9
Fungi	1	*Candida albicans*	227	8.0	2	0.3	0	0.0	2	1.3
	2	Other *Candida* spp.^f^	207	7.3	0	0.0	1	0.8	6	4.0
	Other fungi^g^		10	0.4	0	0.0	0	0.0	0	0.0
	Total fungal		444	15.6	2	0.3	1	0.8	8	5.3
Total			2,854	100	780	100	130	100	151	100

Abbreviations: CSF shunt, cerebrospinal fluid shunt; ICU-CLABSI, intensive care unit central line-associated bloodstream infections

^a^ Frequency distribution percentage rounded to the nearest tenth decimal

^b^ Coagulase-negative staphylococci included *S. lugdunensis*, *S. haemolyticus*, *S. epidermidis*, *S. capitis*, *S. hominis* and *S. warneri*

^c^ *Staphylococcus aureus* includes methicillin-resistant *S. aureus*, methicillin-susceptible *S. aureus* and unspecified *S. aureus*

^d^ Other gram-positive pathogens included anaerobic gram-positive cocci, *Finegoldia magna*, *Clostridioides* spp., *Lactobacillus* spp. and others

^e^ Other gram-negative pathogens included *Stenotrophomonas* spp., *Morganella morganii*, *Proteus mirabilis*, *Pantoea* spp., *Prevotella* spp., *Bacteroides fragilis* and others

^f^ Other *Candida* spp. included *C. dubliniensis*, *C. glabrata*, *C. krusei*, *C. lusitaniae*, *C. parapsilosis* and *C. tropicalis*

^g^ Other fungi included *Aspergillus* spp., *Trichophyton tonsurans* and unspecified fungi

### Intensive care unit central line-associated bloodstream infections

**Infection characteristics:** Between 2019 and 2023, a total of 2,582 CLABSIs were reported. The majority were reported in adult mixed ICUs (65%, n=1,677) and NICUs (19%, n=480), reflecting higher site participation in CLABSI surveillance in these ICU settings. Patient demographics and outcomes for ICU-related CLABSIs are presented in **Table 3**. The median age of patients with CLABSIs in adult ICUs was older in the adult CVICU compared to adult mixed ICUs (*p*<0.001). The majority of those with CLABSIs were male across all ICU settings, ranging from 58% in the PICU to 70% in the adult CVICU. Median days from ICU admission to infection was longest in the PICU (26 days, IQR: 11−65 days) while median days from ICU to infection was 11−14 days in all other ICU settings (*p*<0.001).

**Table 3:** Patient characteristics and outcomes of intensive care unit central line-associated bloodstream infections, 2019–2023

Characteristic	Adult mixed ICU (n=1,677)	Adult CVICU (n=153)	PICU (n=272)	NICU (n=480)
Age, median (IQR)	59 years (46 years, 69 years)	65 years (52 years, 72 years)	7 months (3 months, 36 months)	19 days (9 days, 48 days)
Sex, female, n/N (%)	557/1,677 (33%)	46/153 (30%)	113/272 (42%)	184/480 (38%)
Birthweight (g), median (IQR)	N/A	N/A	N/A	928 (IQR: 670–2,100)
Gestational age (weeks), median (IQR)	N/A	N/A	N/A	27 (IQR: 24–34)
Days from ICU admission to infection, median (IQR)	11 (IQR: 6–22)	11 (IQR: 6–20)	26 (IQR: 11–65)	14 (IQR: 8–35)
Death, thirty-day all cause, n/N (%)	526/1,673 (31%)	50/153 (33%)	26/272 (9.6%)	51/478 (11%)

Abbreviations: CVICU, cardiovascular surgery intensive care unit; ICU, intensive care unit; IQR, interquartile ranges; NICU, neonatal intensive care unit; N/A, not applicable; PICU, paediatric intensive care unit

**Figure 1:**
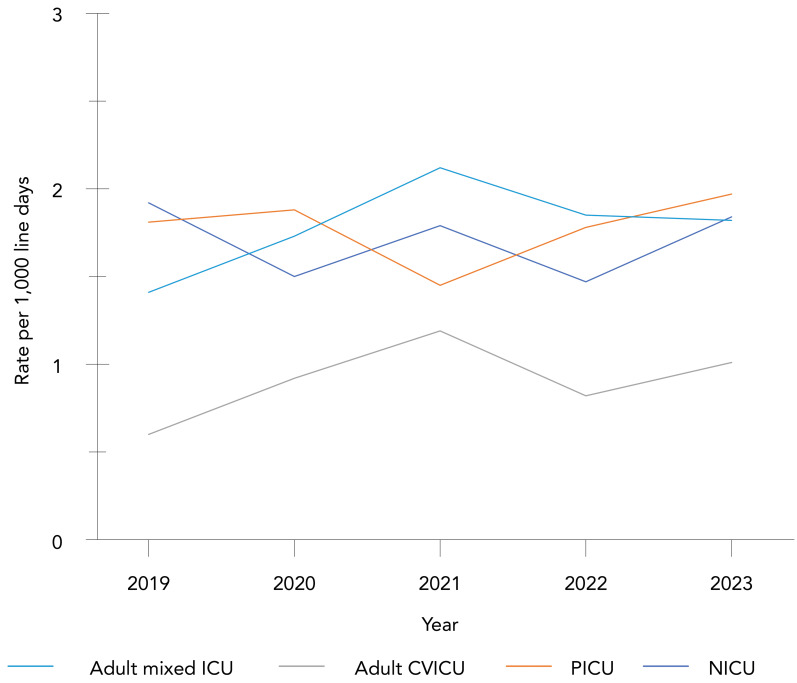
Rate of central line-associated bloodstream infection per 1,000 line days by intensive care unit type, 2019–2023 Abbreviations: CVICU, cardiovascular intensive care unit; ICU, intensive care unit; NICU, neonatal intensive care unit; PICU, paediatric intensive care unit

**Trends over time:** Adult mixed ICUs had the highest CLABSI rates (1.80 infections per 1,000 line days), followed by PICUs (1.79 infections per 1,000 line days), NICUs (1.71 infections per 1,000 line days) and adult CVICUs (0.90 infections per 1,000 line days) (Appendix, **Table A1**). From 2019 to 2023, CLABSIs rates in adult ICU settings fluctuated and increased non-significantly for adult mixed ICUs (29%, 1.41–1.82 infections per 1,000 line days, *p*=0.46) and adult CVICUs (68%, 0.6–1.01 infections per 1,000 line days, *p*=0.46) (**Figure 1**). Adult mixed ICU CLABSI rates peaked at a rate of 2.12 infections per 1,000 line days in 2021 and have since declined. Though rates of CLABSI in adult CVICUs were low overall, a sensitivity analysis among sites that submitted adult CVICU CLABSI data during the entire five-year study period (n=7 hospitals) confirmed a non-significant increase in CLABSI incidence, though less substantial (43%) (data not shown). Catheter utilization from 2019 to 2023 remained stable, ranging from 71%–74% in adult mixed ICUs. In adult CVICUs, catheter utilization was higher compared to adult mixed ICUs, ranging from 80%–87% in adult CVICUs, with an outlier of 66% in 2023 (data not shown).

In paediatric ICUs, NICU and PICU, CLABSI fluctuated from 2019 to 2023, with NICU CLABSI rates ranging between 1.47 to 1.92 infections per 1,000 line days while PICU CLABSIs were lowest in 2021 (1.45 infections per 1,000 lines days), followed by a return to pre-COVID-19 pandemic levels. Catheter utilization in PICUs ranged from 59%–67% from 2019 to 2023 while NICU had the lowest catheter utilization overall during the same time period, ranging from 29%–33%.

All-cause mortality at thirty days was highest in the adult mixed ICU and adult CVICU at 31% and 33%, respectively, while thirty-day all-cause mortality ranged from 9.6%–11% in paediatric and neonatal ICU settings. The most commonly identified pathogens among ICU-CLABSIs overall were CoNS and *Enterococcus* spp. (23.2% and 20.7%, respectively), which aligned with the most commonly identified pathogens among adult mixed ICUs and adult CVICUs. Among PICU and NICU CLABSIs, CoNS and *S. aureus* were the most commonly identified pathogens (data not shown).

### Hip and knee surgical site infections

**Infection characteristics:** Between 2019 and 2023, a total of 694 complex hip and knee SSIs were reported, with hip arthroplasties accounting for the majority of cases (n=432, 62%). Among these SSIs, 51% (n=351) were organ/space infections, while 49% (n=343) were deep incisional infections (**Table 4**). The median patient age was 68 years (IQR: 59–75 years) for hip SSIs and 67 years (IQR: 60–74 years) for knee SSIs. The median time from procedure to infection onset was 22 days (IQR: 15–34 days) for hip SSIs and 24 days (IQR:17–37 days) for knee SSIs. The median length of stay was two days for both hip (IQR: 1–7 days) and knee (IQR: 1–3 days) SSIs.

**Table 4:** Frequency of hip and knee surgical site infections by year and infection type, 2019–2023

Year	Deep incisional SSI		Organ/space SSI		All cases
	n	%	n	%	n
**Hip arthroplasty**					
2019	52	49.5	53	50.5	105
2020	22	44.9	27	55.1	49
2021	44	49.4	45	50.6	89
2022	48	46.2	56	53.9	104
2023	46	54.1	39	45.9	85
Overall	212	49.1	220	50.9	432
**Knee arthroplasty**					
2019	27	50.9	26	49.1	53
2020	14	37.8	23	62.2	37
2021	23	62.2	14	37.8	37
2022	33	54.1	28	45.9	61
2023	34	46.0	40	54.1	74
Overall	131	50.0	131	50.0	262

Abbreviation: SSI, surgical site infection

**Figure 2:**
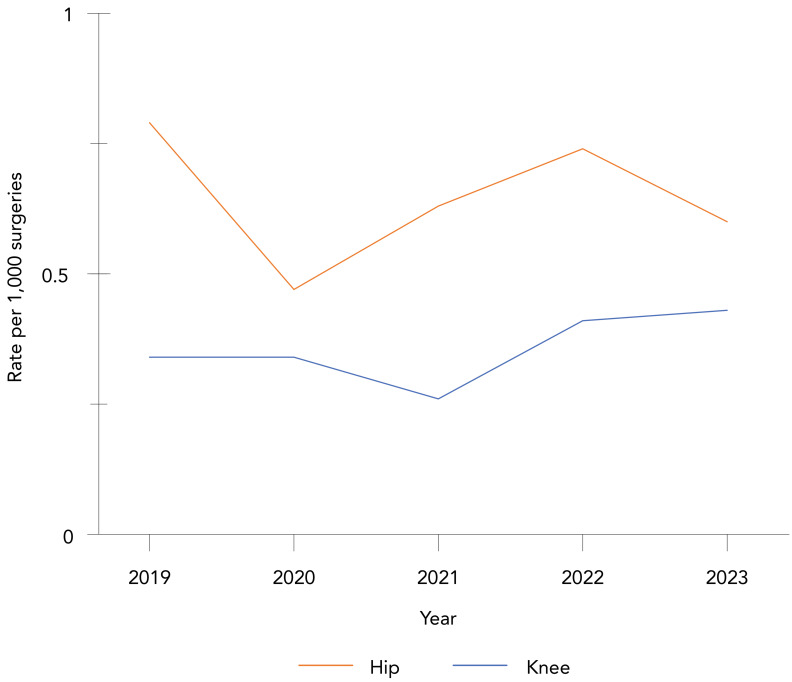
Rate of hip and knee surgical site infections per 100 surgeries, 2019–2023

**Trends over time:** Between 2019 and 2023, knee SSI rates increased non-significantly by 26% (0.34–0.43 infections per 100 surgeries, *p*=0.62), while hip SSI rates fluctuated between 0.47 and 0.79 infections per 100 surgeries (*p*=0.21) (**Figure 2****;** Appendix, **Table A2**). The majority of patients (79%, n=550/692) with a hip or knee SSI were readmitted, and 66% (n=453/685) required revision surgery. Within 30 days after the first positive culture, 14 all-cause deaths (3.3%, n=14/418) were reported among patients with a complex SSI following a hip arthroplasty, while no deaths were reported among knee arthroplasty SSI cases. The most commonly identified pathogens were *S. aureus* (41%) and CoNS (17%), with no significant differences by infection type (data not shown).

### Cerebrospinal fluid shunt surgical site infections

**Infection characteristics:** Between 2019 and 2023, a total of 120 CSF shunt SSIs were reported. The median patient age was 48 years (IQR: 36–61 years) for adult patients and two years (IQR: 0.3–9 years) for paediatric patients. The median time from procedure to infection onset was 19 days (IQR: 10–40 days). More than half of CSF shunt SSIs (56%, n=67/120) were identified from new surgeries, while 44% (n=53/120) were from revision surgeries. Women represented 49% (n=59/120) of cases.

**Figure 3:**
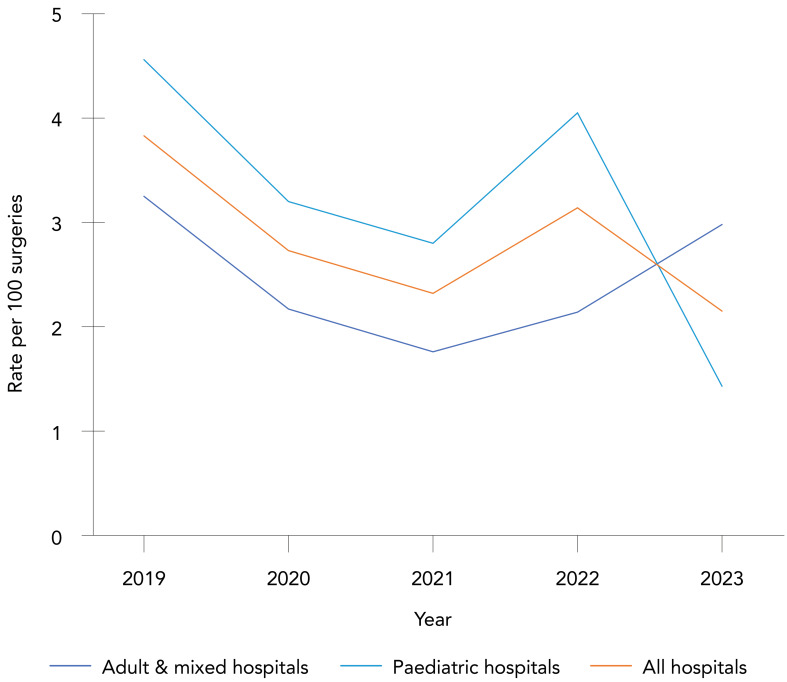
Cerebrospinal fluid shunt surgical site infection rates per 100 surgeries by hospital type^a^, 2019–2023 ^a^ All hospitals include adult, mixed and paediatric hospitals participating in cerebrospinal fluid shunt surgical site infection surveillance

**Trends over time:** The overall rate of CSF shunt SSIs was 2.89 infections per 100 surgeries (range: 2.15–3.83 infections per 100 surgeries, Appendix, **Table A3**). Paediatric and adult/mixed hospitals infection rates were not significantly different at 3.28 and 2.49 infections per 100 surgeries, respectively (*p*=0.15). From 2019 to 2023, no significant trend was observed in CSF shunt SSI rates for adult and mixed hospitals (range: 1.76–3.25 infections per 100 surgeries, *p*=0.40), paediatric hospitals (range: 1.43–4.56 infections per 100 surgeries, *p*=0.11) and all hospital types combined (*p*=0.11) (**Figure 3**). The most commonly identified pathogens from CSF shunt SSIs were CoNS and *S. aureus* (35% and 29% of identified pathogens, respectively). Outcome data were not collected for CSF shunt SSI surveillance.

### Paediatric cardiac surgical site infections

**Infection characteristics:** Between 2019 and 2023, a total of 184 paediatric cardiac SSIs were reported (**Table 5**). The majority of infections were superficial incisional SSIs (65%), followed by organ/space infections (26%) and deep incisional infections (9%). The median patient age was 63 days (IQR: 7–347 days), and the median time from surgery to infection onset was 15 days (IQR: 8–23 days). The proportion of deep incisional infections increased from 5.7% in 2019 to 15% in 2023, though this trend was not significant (*p*=0.09, Table 5).

**Table 5:** Paediatric cardiac surgical site infection rates by year and infection type, 2019–2023

Year	Superficial incisional SSI cases		Organ/space SSI cases		Deep incisional SSI cases		All cases^a^
	n	%	n	%	n	%	
2019	19	54.3%	14	40.0%	2	5.7%	35
2020	29	78.4%	6	16.2%	2	5.4%	37
2021	23	65.7%	9	25.7%	3	8.6%	35
2022	16	64.0%	6	24.0%	3	12.0%	25
2023	32	61.5%	12	23.1%	8	15.4%	52
Overall	119	64.7%	47	25.5%	18	9.8%	184

Abbreviation: SSI, surgical site infection

^a^ Excludes cases with missing infection type information

**Figure 4:**
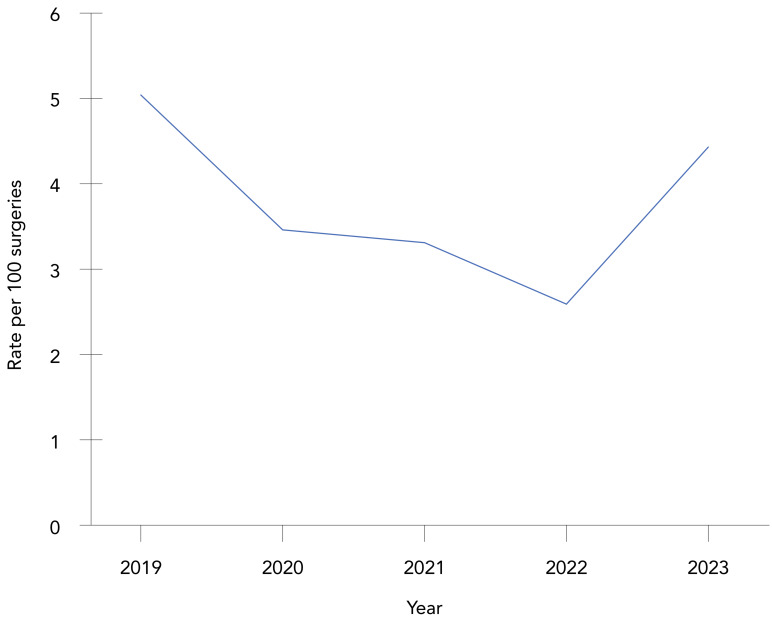
Paediatric cardiac surgical site infection rates per 100 surgeries, 2019–2023

**Trends over time:** The overall paediatric cardiac SSI rate was 3.7 infections per 100 surgeries, with annual rates fluctuating between 2.59 and 5.04 infections per 100 surgeries (**Figure 4****;** Appendix, **Table A4**). No significant trend was observed during this five-year period. At 30 days post-infection, 71% of patients had been discharged. Five deaths (2.7% of cases) were reported within 30 days of infection onset, including two deaths directly attributable to the paediatric cardiac SSI. The most commonly identified pathogens were *S. aureus* (61%) and CoNS (13%), with no differences observed by infection type (data not shown).

### Antibiogram

**Figure 5:**
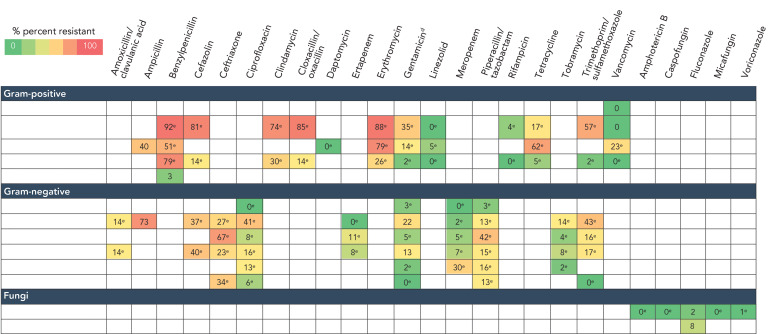
Antibiogram results^a^ from pathogens identified from intensive care unit central line-associated bloodstream infections, 2019–2023^b,c,d,e^ ^a^ Antibiotic/organism combinations with fewer than 30 tests were excluded ^b^ Coagulase-negative staphylococci included *S. lugdunensis, S. haemolyticus, S. epidermidis, S. capitis, S. hominis* and *S. warneri* ^c^ Included methicillin-susceptible *S. aureus* and methicillin-resistant *S. aureus* (MRSA) ^d^ Gentamicin synergy for gram-positive organisms ^e^ Less than 90% of isolates were tested

**Figure 6:**
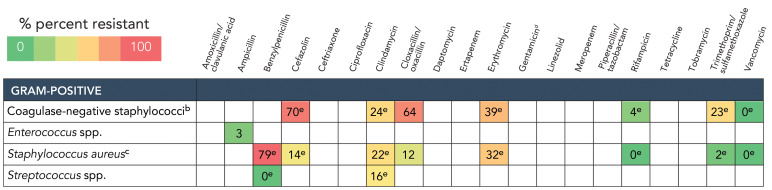
Antibiogram results^a^ from pathogens identified from hip and knee, cerebrospinal fluid shunt and paediatric cardiac surgical site infections, 2019–2023^b,c,d,e^ ^a^ Antibiotic/organism combinations with fewer than 30 tests were excluded ^b^ Coagulase-negative staphylococci included *S. lugdunensis*, *S. haemolyticus*, *S. epidermidis*, *S. capitis*, *S. hominis* and *S. warneri* ^c^ Included methicillin-susceptible *S. aureus* and methicillin-resistant *S. aureus* (MRSA) ^d^ Gentamicin synergy for gram-positive organisms ^e^ Less than 90% of isolates were tested

Results of antimicrobial susceptibility testing for the most frequently identified gram-positive, gram-negative and fungal pathogens from device and surgical procedure-related HAIs are listed in **Figure 5** and **Figure 6**. The *S. aureus* isolates were resistant to cloxacillin/oxacillin (MRSA) in 14% (n=27/198) of ICU-CLABSIs and 14% (n=46/337) of SSIs. Meropenem resistance ranged from 0% to 30% in gram-negative pathogens identified from ICU-CLABSIs. No meropenem resistance was observed among pathogens isolated from SSIs. Eighty-three vancomycin-resistant *Enterococci* were identified among ICU-CLABSIs (23%, n=335).

## Discussion

This report summarizes 2,582 device-related infections and 1,029 surgical procedure-related infections as well as antibiogram data identified over five years of surveillance (2019–2023) from 68 hospitals across Canada. During this time, rates of device and surgical procedure-related HAIs have increased non-significantly by 26% for knee SSIs while ICU-CLABSIs have fluctuated and increased in all ICU settings throughout the study period except for in the NICU.

### Intensive care unit central line-associated bloodstream infections

Central line-associated bloodstream infections were the predominant HAIs in this surveillance report, with observed case counts more than double those of all SSIs combined. Overall rates of CLABSIs in adult ICUs from 2019 to 2023 (1.80 and 0.90 infections per 1,000 line days in adult mixed ICUs and CVICUs, respectively) were lower than those reported in England during the same period (ranging from 1.7 to 3.3 infections per 1,000 line days) ((10)). Conversely, CNISP reported higher ICU-CLABSI rates than southeast Australia over the same timeframe, ranging from 0.57 to 0.85 infections per 1,000 line days ((11)). Standardized infection ratios (defined as the ratio of observed number of infections compared to the 2015 baseline) from the United States have reported a 23% (95% CI: 21%–25%) decrease in 2023 CLABSI incidence across all ICU locations, with a 40% (95% CI: 36%–43%) decrease in CLABSIs noted in the NICU ((12)). According to CNISP-reported data in Canada, the incidence of CLABSIs in paediatric and neonatal ICUs was higher compared to England, where rates decreased from 2019 to 2023 and remained lower overall (0.95 and 1.26 infections per 1,000 line days, respectively) ((10)).

### Surgical site infections

**Hip and knee surgical site infections:** Among SSIs included in this surveillance report, hip and knee SSIs were the most prevalent. Hip SSI rates fluctuated across reporting years, while knee SSI rates increased non-significantly. Over the same time period (2018–2023), surveillance from the United Kingdom showed a similar knee SSI rate to the CNISP data (0.4 infections per 100 procedures), while knee SSI rates in the United Kingdom were slightly lower than CNISP data (0.5 infections per 100 procedures vs. 0.65 infections per 100 procedures) ((13)). Hip and knee SSI rates in Southern Australia were higher overall than CNISP data and have also seen increases in recent years; hip SSI rates increased from 2018 to 2020 (1.80–1.91 infections per 100 procedures), while knee SSI rates increased from 0.79 to 0.88 infections per 100 procedures, during the same time period ((14)). The most common pathogens among hip and knee SSIs were *S. aureus* and CoNS, in accordance with results from other regions.

**Cerebrospinal fluid shunt surgical site infections:** The overall rate of SSIs from CSF shunts was 2.89 per 100 surgeries from 2019 to 2023. A national survey conducted in England in 2017 reported a mean brain shunt infection rate of 1.9% (range: 0%–4.4%), which is lower than the overall rate reported by CNISP ((15)). In contrast, a retrospective single-center study in Sweden reported a higher shunt infection rate of 4.8% in adult hydrocephalus patients who underwent surgery between 2013 and 2019 ((16)). Due to the lack of recent literature, comparisons with other regions are limited. To address this, we compared 2019–2023 data with historical CNISP surveillance data from 2011 to 2020 (17). Consistent with this earlier data, we observed a fluctuating trend in CSF shunt SSI rates from 2019 to 2023 ((17)). Stratification of CSF shunt SSI data by paediatric and adult/mixed hospitals showed that, from 2019 to 2023, paediatric rates (3.28 infections per 100 surgeries) and adult rates (2.49 infections per 100 surgeries) were not significantly different. Among paediatric patients, CSF shunt SSI rates from 2019 to 2023 (3.3%) were lower than those reported from 2000 to 2002 (4.9%), indicating a decline in SSI rates in this population compared to historical data ((18)). Similarly, the CSF shunt SSI rate among adult patients from 2019 to 2023 (2.5%) was lower than the rate reported from 2000 to 2002 (3.2%) ((18)).

**Paediatric cardiac surgical site infections:** Overall, from 2019 to 2023, 3.7 paediatric cardiac SSIs were reported per 100 surgeries. Data reported through the CNISP network indicated no significant trend in paediatric cardiac SSI rates from 2019 to 2023. Due to the lack of recent literature available, the ability to compare these results to other regions is limited; however, one academic medical centre in California reported a reduction in paediatric cardiac SSI rates from 3.4 SSIs per 100 surgeries in 2013 to 0.9 SSIs per 100 surgeries in 2017 following the implementation of a post-operative SSI reduction care bundle ((19)). In contrast, the United States reported a standardized infection ratio (defined as the ratio of observed number of infections compared to the predicted number of infections) of 0.71 (95% CI: 0.56–0.90) in 2023 when compared to the 2015 baseline period, indicating a decrease in paediatric cardiac SSI rates ((12)). However, only deep incisional and organ/space SSIs were included in these calculations ((20)).

### Antibiogram

Due to the lack of recent literature, the ability to compare these results with other regions is limited. To address this, we compared 2019–2023 data with the previous 2018–2022 surveillance report; however, since the time periods overlap, observed changes may not reflect true trends and should be interpreted with caution ((21)). The percentage of *S. aureus* isolates that were MRSA among ICU-CLABSIs (14%) and SSIs (11%) in the CNISP network remained relatively stable over the 2019–2023 period compared to previous surveillance data from 2018–2022, where MRSA accounted for 15% of ICU-CLABSIs and 12% of SSIs. Among *Enterococcus* spp. identified in ICU-CLABSIs, 23% were vancomycin-resistant *Enterococci*, consistent with earlier findings ((21)). Similarly, meropenem resistance among gram-negative pathogens identified in ICU-CLABSIs remained highest in *Pseudomonas* spp. (30%), while resistance in other gram-negative pathogens ranged from 0% to 7% ((21)). Notably, meropenem resistance in *Pseudomonas* spp. identified in ICU-CLABSIs decreased from 38% in 2018–2022 to 30% in 2019–2023 ((21)).

### Strengths and limitations

The main strength of CNISP surveillance is the standardized collection of detailed epidemiological and molecular linked data from a large representative network of sentinel hospitals across Canada. From 2019 to 2023, CNISP coverage of Canadian acute care beds has increased from 33% to 37%, including increased representativeness in northern, community, rural and Indigenous populations. To further improve representativeness, CNISP has launched a simplified dataset accessible to all acute care hospitals across Canada to collect and visualize annual HAI rate data. The number of hospitals participating in each HAI surveillance project differed and epidemiologic data collected were limited to the information available in the patient charts. For CLABSI surveillance, data were limited to infections occurring in the ICU settings, and as such may only represent a subset of CLABSIs occurring in the hospital. Further, when comparing our infection rates with data from other countries, several limitations must be considered, including differences in surveillance methodologies, patient populations, and the number and types of hospitals under surveillance.

## Conclusion

This report provides an updated summary of rates, pathogen distributions and antimicrobial resistance patterns among select device and surgical procedure-related HAIs and relevant pathogens. The collection and analysis of national surveillance data are important to understanding and reducing the burden of device and surgical procedure-related HAIs. These data provide benchmark rates for national and international comparison and inform antimicrobial stewardship and infection prevention and control programs and policies.

## Appendix

### Case definitions

Central line-associated bloodstream infection

Only central line-associated bloodstream infections (CLABSIs) related to an intensive care unit (ICU) admission were included in surveillance.

Bloodstream infections case definition:

Bloodstream infection is **NOT** related to an infection at another site and it meets one of the following criteria:

**Criterion 1:** Recognized pathogen cultured from at least one blood culture, unrelated to infection at another site.

OR

**Criterion 2:** At least one of: fever (higher than 38°C core), chills, hypotension; if aged younger than 1 year, fever (higher than 38°C core), hypothermia (lower than 36°C core), apnea or bradycardia **AND** common skin contaminant (see list below) cultured from at least two blood cultures drawn on separate occasions or at different sites, unrelated to infection at another site. Different sites may include peripheral veins, central venous catheters or separate lumens of a multilumen catheter. Different times include two blood cultures collected on the same or consecutive calendar days via separate venipunctures or catheter entries. The collection date of the first positive blood culture is the date used to identify the date of positive culture. Two positive blood culture bottles filled at the same venipuncture or catheter entry constitute only one positive blood culture.

Central line-associated bloodstream infection case definition:

A CLABSI must meet one of the following criteria:

**Criterion 1:** A laboratory-confirmed bloodstream infection (LCBSI) where a central line catheter (CL) or umbilical catheter (UC) was in place for more than two calendar days on the date of the positive blood culture, with day of device placement being Day 1.

OR

**Criterion 2:** A LCBSI where a CL or UC was in place more than two calendar days and then removed on the day or one day before positive blood culture was drawn.

Intensive care unit-related central line-associated bloodstream infection case definition:

A CLABSI related to an ICU if it meets one of the following criteria:

**Criterion 1:** CLABSI onset after two days of ICU stay.

OR

**Criterion 2:** If the patient is discharged or transferred out of the ICU, the CLABSI would be attributable to the ICU if it occurred on the day of transfer or the next calendar day after transfer out of the ICU.

Note: If the patient is transferred into the ICU with the CL and the blood culture was positive on the day of transfer or the next calendar day, then the CLABSI would be attributed to the unit where the line was inserted.

Common skin contaminants:

Diphtheroids, *Corynebacterium* spp., *Bacillus* spp., *Propionibacterium* spp., coagulase-negative staphylococci (including *S. epidermidis*), viridans group streptococci, *Aerococcus* spp., *Micrococcus* spp. and *Rhodococcus* spp.

### Hip and knee surgical site infection

Only complex surgical site infections (SSIs) (deep incisional or organ/space) following hip and knee arthroplasty were included in surveillance.

A deep incisional surgical site infection must meet the following criterion:

Infection occurs within 90 days after the operative procedure and the infection appears to be related to the operative procedure and involves deep soft tissues (e.g., facial and muscle layers) of the incision and the patient has at least **ONE** of the following:

· Purulent drainage from the deep incision but not from the organ/space component of the surgical site

· Deep incision that spontaneously dehisces or is deliberately opened by the surgeon and is culture-positive or not cultured when the patient has at least one of the following signs or symptoms: fever (higher than 38°C) or localized pain or tenderness (a culture-negative finding does not meet this criterion)

· An abscess or other evidence of infection involving the deep incision is found on direct examination, during reoperation or by histopathologic or radiologic examination

· Diagnosis of a deep incisional SSI by a surgeon or attending physician

An organ/space surgical site infection must meet the following criterion:

Infection occurs within 90 days after the operative procedure and the infection appears to be related to the operative procedure and infection involves any part of the body, excluding the skin incision, fascia or muscle layers, that is opened or manipulated during the operative procedure and patient has at least **ONE** of the following:

· Purulent drainage from a drain that is placed through a stab wound into the organ/space

· Organisms isolated from an aseptically obtained culture of fluid or tissue in the organ/space

· An abscess or other evidence of infection involving the organ/space that is found on direct examination, during reoperation or by histopathologic or radiologic examination

· Diagnosis of an organ/space SSI by a surgeon or attending physician

### Cerebrospinal fluid shunt surgical site infection

Only patients who underwent a placement or revision of a cerebrospinal fluid (CSF) shunting device and the infection occurred within 90 days of surgery were included in surveillance.

Cerebrospinal fluid shunt-associated surgical site infection case definition:

An internalized CSF shunting device is in place **AND** a bacterial or fungal pathogen(s) is identified from the CSF **AND** is associated with at least **ONE** of the following:

· Fever (temperature 38°C or higher)

· Neurological signs or symptoms

· Abdominal signs or symptoms

· Signs or symptoms of shunt malfunction or obstruction

### Paediatric cardiac surgery surgical site infection

Only surgical site infections following open-heart surgery with cardiopulmonary bypass among paediatric patients (younger than 18 years of age) were included in surveillance.

A **superficial incisional SSI** must meet the following criterion: Infection occurs within 30 days after the operative procedure and involves only skin and subcutaneous tissue of the incision and meets at least **ONE** of the following criteria:

· Purulent drainage from the superficial incision

· Organisms isolated from an aseptically obtained culture of fluid or tissue from the superficial incision

· At least **ONE** of the following signs or symptoms of infection:

o Pain or tenderness, localized swelling, redness or heat, and the superficial incision is deliberately opened by a surgeon, and is culture-positive or not cultured (a culture-negative finding does not meet this criterion)

o Diagnosis of superficial incisional SSI by the surgeon or attending physician

A **deep incisional SSI** must meet the following criterion:

Infection occurs within 90 days after the operative procedure and the infection appears to be related to the operative procedure **AND** involves deep soft tissues (e.g., facial and muscle layers) of the incision **AND** the patient has at least **ONE** of the following:

· Purulent drainage from the deep incision but not from the organ/space component of the surgical site

· Deep incision spontaneously dehisces or is deliberately opened by the surgeon and is culture-positive or not cultured when the patient has at least one of the following signs or symptoms: fever (higher than 38°C) or localized pain or tenderness (a culture-negative finding does not meet this criterion)

· An abscess or other evidence of infection involving the deep incision is found on direct examination, during reoperation or by histopathologic or radiologic examination

· Diagnosis of a deep incisional SSI by a surgeon or attending physician

An **organ/space SSI** must meet the following criterion:

Infection occurs within 90 days after the operative procedure and the infection appears to be related to the operative procedure **AND** infection involves any part of the body, excluding the skin incision, fascia or muscle layers, that is opened or manipulated during the operative procedure **AND** the patient has at least **ONE** of the following:

· Purulent drainage from a drain that is placed through a stab wound into the organ/space

· Organisms isolated from an aseptically obtained culture of fluid or tissue in the organ/space

· An abscess or other evidence of infection involving the organ/space that is found on direct examination, during reoperation or by histopathologic or radiologic examination

**Table A1:** Rate of central line-associated bloodstream infection per 1,000 line days by intensive care unit type, 2019–2023

Year	Adult mixed ICU	Adult CVICU	NICU	PICU
2019	1.41	0.6	1.81	1.92
2020	1.73	0.92	1.88	1.5
2021	2.12	1.19	1.45	1.79
2022	1.85	0.82	1.78	1.47
2023	1.82	1.01	1.97	1.84
Overall	1.80	0.90	1.79	1.71

Abbreviations: CVICU, cardiovascular intensive care unit; ICU, intensive care unit; NICU, neonatal intensive care unit; PICU, paediatric intensive care unit

**Table A2:** Rate of hip and knee surgical site infections per 100 surgeries, 2019–2023

Year	Hip	Knee
2019	0.79	0.34
2020	0.47	0.34
2021	0.63	0.26
2022	0.74	0.41
2023	0.60	0.43
Overall	0.65	0.36

**Table A3:** Cerebrospinal fluid shunt surgical site infection rates per 100 surgeries by hospital type, 2019–2023

Year	Adult and mixed hospitals	Paediatric hospitals	All hospitals^a^
2019	3.25	4.56	3.83
2020	2.17	3.2	2.73
2021	1.76	2.80	2.32
2022	2.14	4.05	3.14
2023	2.98	1.43	2.15
Overall	2.49	3.28	2.89

^a^ All hospitals include adult, mixed, and paediatric hospitals participating in cerebrospinal fluid shunt surgical site infection surveillance

**Table A4:** Paediatric cardiac surgical site infection rates per 100 surgeries, 2019–2023

Year	Rate
2019	5.04
2020	3.46
2021	3.31
2022	2.59
2023	4.43
Overall	3.71
